# Genes with Relevance for Early to Late Progression of Colon Carcinoma Based on Combined Genomic and Transcriptomic Information from the Same Patients

**DOI:** 10.4137/cin.s4545

**Published:** 2010-04-23

**Authors:** Kristina K. Lagerstedt, Erik Kristiansson, Christina Lönnroth, Marianne Andersson, Britt-Marie Iresjö, Annika Gustafsson, Elisabeth Hansson, Ulf Kressner, Svante Nordgren, Fredrik Enlund, Kent Lundholm

**Affiliations:** 1 Department of Surgery, Institute of Clinical Sciences, Sahlgrenska Academy, Sahlgrenska University Hospital, Gothenburg, Sweden; 2 Department of Zoology, University of Gothenburg, Sweden; 3 Department of Surgery, Uddevalla Hospital, Uddevalla, Sweden; 4 Department of Clinical Chemistry, Sahlgrenska Academy, Sahlgrenska University Hospital, Gotenhburg, Sweden. Email: kent.lundholm@surgery.gu.se

**Keywords:** CGH array, colorectal cancer, microRNA, transcription

## Abstract

**Background:**

Genetic and epigenetic alterations in colorectal cancer are numerous. However, it is difficult to judge whether such changes are primary or secondary to the appearance and progression of tumors. Therefore, the aim of the present study was to identify altered DNA regions with significant covariation to transcription alterations along colon cancer progression.

**Methods:**

Tumor and normal colon tissue were obtained at primary operations from 24 patients selected by chance. DNA, RNA and microRNAs were extracted from the same biopsy material in all individuals and analyzed by oligo-nucleotide array-based comparative genomic hybridization (CGH), mRNA- and microRNA oligo-arrays. Statistical analyses were performed to assess statistical interactions (correlations, co-variations) between DNA copy number changes and significant alterations in gene and microRNA expression using appropriate parametric and non-parametric statistics.

**Results:**

Main DNA alterations were located on chromosome 7, 8, 13 and 20. Tumor DNA copy number gain increased with tumor progression, significantly related to increased gene expression. Copy number loss was not observed in Dukes A tumors. There was no significant relationship between expressed genes and tumor progression across Dukes A–D tumors; and no relationship between tumor stage and the number of microRNAs with significantly altered expression. Interaction analyses identified overall 41 genes, which discriminated early Dukes A plus B tumors from late Dukes C plus D tumor; 28 of these genes remained with correlations between genomic and transcriptomic alterations in Dukes C plus D tumors and 17 in Dukes D. One microRNA (microR-663) showed interactions with DNA alterations in all Dukes A-D tumors.

**Conclusions:**

Our modeling confirms that colon cancer progression is related to genomic instability and altered gene expression. However, early invasive tumor growth seemed rather related to transcriptomic alterations, where changes in microRNA may be an early phenomenon, and less to DNA copy number changes.

## Introduction

Overwhelming amount of information is appearing in the literature on genetic alterations associated to invasive colorectal cancer.^1^ It is so far unclear to what extent such findings are primary causes of neoplastic transformation and tumor progression or may rather represent events secondary to genetic instability. Unfortunately, it would require hundred thousands of patients with defined colon cancer disease and controlled follow up to discriminate and validate both genetic and epigenetic information by traditional multivariate analyses.^2^–^5^ This fact became evident to us in previous evaluations of results based on genome wide DNA alterations in progressive colon cancer based on BAC CGH analyses in patients with different survival,^6^ as also emphasized by others.^7^ Thus, it seems practically impossible to rank appearing DNA sequence alterations in relationship to progressive disease and clinical outcome, accounting for defined and undefined standard elements including epigenetics.^3^,^4^ A major part of correlates and relationships may after all only represent indirect or secondary phenomena to underlying critical cellular events despite sufficient statistical power or information on complete genome wide alterations.^8^,^9^ Therefore, simplistic models are required as alternatives to traditional statistics in order to efficiently screen for and suggest candidate DNA regions of primary importance for appearing invasive growth and subsequent progression of colorectal cancer. In line with this speculation we found it interesting to relate significant DNA copy number changes to either significantly changed gene expressions or posttranscriptional control of RNA in tumor biopsies from colon cancer; all processed from the same patients. The present study provides such in silico analyses on well defined and quality controlled tumor material from selected patients with colorectal cancer of Dukes A, B, C and D tumor stage as surrogate markers for clinical outcome, in order to filter genes within regions with copy number gain and loss by statistical modeling in limited number of patients.^1^

## Materials and Methods

### Patients and clinical details

Intentionally, the patient material comprised a limited number of patients (n = 24) operated on for primary colon carcinoma at Uddevalla Hospital, Sweden between 2001–2003 (Table 1). These patients were selected by chance from a cohort of 486 consecutive patients with colorectal cancer to represent 6 patients, with tumor stage Dukes A, B, C and D, respectively. (Modified Dukes A–D stages correspond to TNM I–IV in present histopathological evaluations). Dukes D tumors were all diagnosed at operations and subsequent histopathological staging. Patient selection was also dependent on the presence of a particular surgeon, patient acceptance to take part in the study, quality control of tissue extracted RNA and the absence of any pharmacological preoperative treatment deemed of importance for the investigation. Thus, none of the patients had experienced any additional specific treatment beside surgery at the time of operation. Patients with rectal and very low sigmoidal tumors were not considered for inclusion. There was no overall difference between the patients when grouped according to Dukes A, B, C and D stages, considering gender and tumor location (Table 1), but Dukes D patients were younger as also observed in the entire cohort of 486 patients (*P* < 0.05). Six patients for each Dukes group were finally available according to above mentioned criteria considering a comparatively even distribution of patient characteristics and disease stage.

### Tissue samples and extraction of DNA and RNA

Biopsies from primary tumors and normal colon tissue were collected from each patient at operation, snap frozen in liquid nitrogen and stored at −80°C. Tissue biopsies were crusched in a mortar and two aliquotes of powdered tissue were used for DNA and total RNA extraction respectively. Genomic DNA and total RNA were from the same tissue source in each patient. DNA was extracted with QIamp DNA mini kit (Qiagen) according to instructions and total RNA was extracted with mirVana total RNA isolation kit (Ambion/Applied Biosystems). All material was quantified by NanoDrop ND-1000 spectrophotometry (NanoDrop Technologies) and total RNA samples were run in Bioanalyzer (Agilent Technologies) to confirm appropriate quality. mRNA expression arrays and DNA on oligo CGH arrays were run in triplicate. MicroRNA expression arrays were run in duplicate (167 or 307 ng DNA depending on array format, 33 ng RNA and 20 ng microRNA were used from each patient). Tumor tissue comprised around 80% malignant cells.^6^

### CGH analysis

Genomic DNA from tumor and normal colon tissue from the 24 patients was separately pooled for analyses with 6 patients in each group according to Dukes A–D. Hybridization of tumor versus normal colon DNA was performed in competition to either 44 K Whole Human Genome oligo arrays (Design 013282, Agilent Technologies) or 4 × 44 K Whole Human Genome oligo arrays (Design 014950, Agilent Technologies). Pooled DNA (1.84 μg/array) for 44 K arrays was labeled with Agilent Genomic DNA Labeling Kit PLUS, hybridized and washed using Agilent Human Genome CGH Microarray Kit 44B and for 4 ×44 K arrays by labeling (1μg DNA/array) with Agilent Genomic DNA Labeling Kit PLUS, hybridized with Agilent Oligo aCGH Hybridization Kit and washed with Agilent Oligo Wash Buffer 1 and 2 set. All labeled samples were checked by NanoDrop spectrophotometry prior to hybridization and arrays were scanned (Agilent scanner G2565 AA, Agilent Technologies).

Analyses of scanned images from CGH two-color oligonucleotide arrays were performed in Feature Extraction 9.1.3.1 (Agilent Technologies). Feature Extraction result files were imported into the statistical language R 2.7.2^10^ where both channels were normalized using median normalization implemented in the Bioconductor package^11^ LIMMA. The technical replicates were averaged and then segmented by DNA copy package using the CBS algorithm with default parameter values.^12^ Minimal common regions (MCR, defined in)^13^ between the different Dukes types were identified using the cghMCR package.^13^ Briefly, gained and lost regions were defined as segment of contiguous probes that showed log_2_ values above or below a cut-off level, defined as one standard deviation of the probe variation calculated from all of the arrays. The cut-off values for both gained and lost segments were estimated to 0.1 (log_2_), which corresponded approximately to the 20th and 80th percentiles of the segment alteration values respectively.^12^

### mRNA expression analysis

Total RNA from tumor and normal tissue was separately pooled as described for CGH analyses; 200 ng of pooled total RNA was labeled with Agilent Two-Color RNA Spike-In Kit (Agilent Technologies), linearly amplified and synthesized to cRNA. Labeled products were checked in a NanoDrop and further hybridized in competition to Agilents Whole Human Genome Oligo Microarrays (Design 014850) with Gene Expression Hybridization Kit (Agilent Technologies). Arrays were washed with Gene Expression Wash Buffer Kit (Agilent Technologies) and scanned (Agilent scanner, Agilent Technologies).

Analyses of scanned images from two-color mRNA expression were performed in Feature Extraction 9.1.3.1 (Agilent Technologies). Feature Extraction result files were imported into the statistical language R 2.7.2 where replicated probes were averaged. 10 Each array was then normalized using Lowess normalization implemented in the Bioconductor package LIMMA.^11^,^14^ A moderated t-statistic, based on an empirical Bayes model were calculated for each gene and the corresponding *p*-value was adjusted for multiple testing using the Benjamini-Hochberg False Discovery Rate (FDR).^15^,^16^ Absolute log fold-change of 1 and FDR of 0.05 were used as cut-off for subsequent analyses. Trends in mRNA expression according to the Dukes types were tested with linear regression within the empirical Bayes model.^15^

### microRNA expression analysis

Total RNA from tumor and normal colon tissue was separately pooled as described; 120 ng of pooled total RNA was labeled with Agilent Cyanine 3-pCp reagent for direct labeling by Agilent microRNA Labeling Reagent and Hybridization Kit (Agilent Technologies). Labeled products were hybridized to Agilent Human microRNA single color microarrays (G4470A, Agilent Technologies, with 470 human, 64 viral probes), washed and scanned on an Agilent scanner. Analyses of scanned images from single-color microRNA expression were performed in Feature Extraction 9.5 (Agilent Technologies). The one-channel Feature Extraction 9.5 result files were imported into R. Identical probes were averaged and the data normalized using quantile-quantile normalization implemented in the Bioconductor R-package Affy.^17^ As for the mRNA expression data, a moderated t-statistic was calculated for each microRNA as well as a *p*-value and the FDR. Cut-off values used in subsequent analyses were an absolute log fold-change of 0.5 and an FDR of 0.05.

### Statistics and mathematical interactions

Group analyses were performed by t-testing or ANOVA and frequency analysis by χ^2^. Statistical interaction analyses (correlations, co-variations, significant alterations) were based on *DNA segments* with copy number changes and significant alterations in expression of defined transcripts. Statistical interactions between altered DNA sequences and mRNA/microRNA expression for a specific region were calculated as follow: First, probes from the microarray were mapped to NCBI Entrez (build 18) genes within the region. The proportion of differentially expressed genes was compared to the entire genome and enrichment was then tested using Fisher’s exact test. The test of interactions was performed for all significant DNA alterations over the entire genome, each chromosome and each aberrant segment according to CGH analysis. Significant correlations between DNA events present in Dukes A plus B tumors versus findings in Dukes C plus D tumors in combination with altered expressions were regarded candidate DNA sequences, that may explain tumor progression. *P* < 0.05 was regarded statistically significant in twotailed tests.

## Results

### DNA alterations

**Figure 1.**
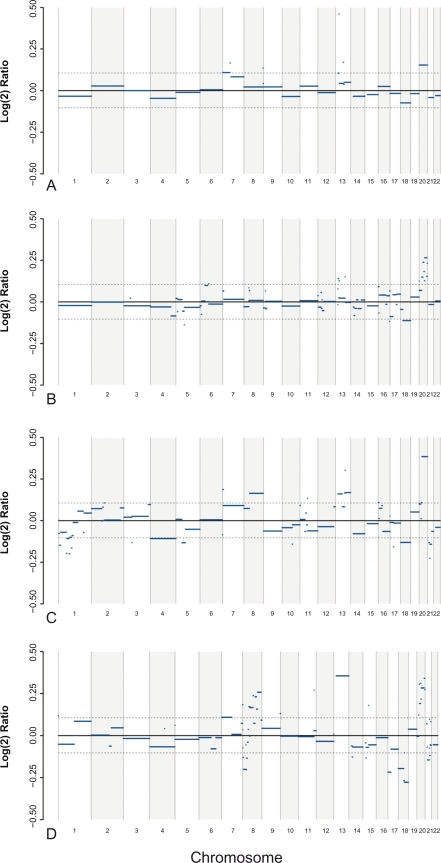
Genome wide overview of DNA segments with sequence variations across chromosomes in Dukes A, B, C and D. Solid lines outside or close to the confidence interval (dashed lines) suggest significant DNA sequence alterations.

**Figure 2.**
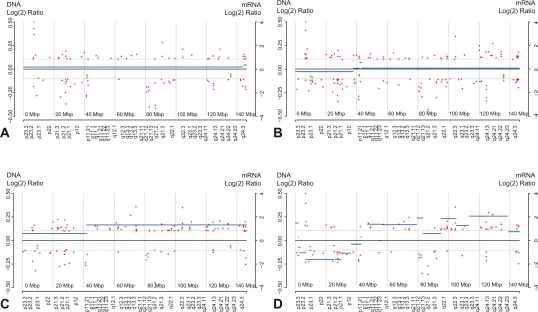
DNA copy number changes on chromosome 8 in Dukes A, B, C and D tumors. Significantly altered DNA segments are indicated by solid lines. Genes with significantly altered expression are indicated by red (mRNA) and green (microRNAs). Dashed lines indicate thresholds for statistically significant DNA segment alterations.

Tumor tissue vs. normal colon tissue Significant tumor DNA copy number changes increased with tumor progression defined as early (Dukes A plus B) versus late tumors (Dukes C plus D) (Fig. 1, Fig. 2, Table 2). Dukes A, B, C, and D tumors displayed DNA alterations in 4%, 4%, 21% and 16% respectively of the entire genome compared to normal colon tissue (*P* < 0.05) (Table 2). Four chromosomes displayed alterations in Dukes A, 6 in Dukes B, 15 in Dukes C and 14 in Dukes D (X and Y excluded). Copy number loss was not observed in Dukes A. Early stage DNA alterations were gain on chromosome 7p, 13q, 20p/q and loss on 18q. Late stage alterations were gain on 7p/q, 8q, 13q, 20p/q and loss on 8p, 17p/q, 18p/q and 21q.

Chromosomes 1–11, 13–18 and 20–21 showed 102 Minimal Common Regions (MCRs) in Dukes A, B, C and D tumors; 78% represented gains and 22% lost regions (not shown). These aberrations equalized 30% of the entire genome (X and Y chromosomes excluded);. 14% of aberrant bases covered by MCR regions were altered in at least 3 out of 4 Dukes groups when analyzed in iterated combinations (ABCD, ABC, ACD, or BCD). These alterations were mainly located on chromosomes 7, 13, 18 and 20. Chromosomes 13 (1 Mb) and 20 (41 Mb) showed gains in all Dukes A–D tumors; 55% of MCRs were found in Dukes A and B tumors and may be considered most relevant for carcinogenesis and early tumor progression. Overall 75% of the MCRs were found in Dukes C and D tumors (not shown).

### mRNA expression

#### Tumor tissue vs. normal colon tissue

**Figure 3.**
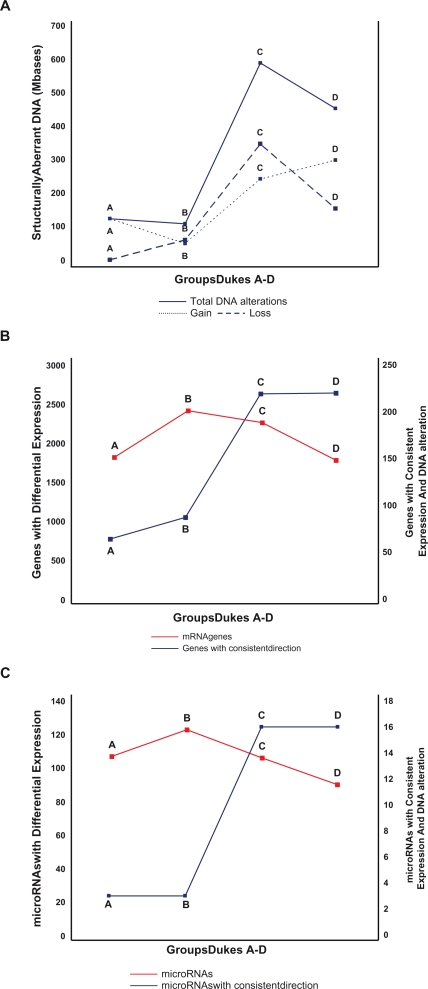
**A**) Distribution of aberrant DNA copy numbers across Dukes A, B, C and D tumors (solid line), DNA loss (dashed line) and gained (semidashed line). **B**) Distribution of significantly changed transcript expression across Dukes A-D tumors (red line) including significant interactions between DNA and RNA alterations (solid line). **C**) Distribution of significantly altered microRNAs across Dukes A–D tumors (red line) including significant interactions between DNA and microRNA alterations (solid line).

Distribution of genes with altered expression among Dukes A–D tumors is summarized in Figure 3b and Table 3. There was no significant relationship between the number of expressed genes and tumor progression (Fig. 3b). Six, 8, 8 and 6 percent of all genes showed significantly altered expression (FC > 1, FDR < 0.05) in tumor tissue compared to normal colon tissue in Dukes A, B, C and D respectively. Downregulation was more common than upregulation in Dukes A and B tumors (*P* < 0.05), without any such difference between up and downregulation in Dukes C and D tumors. Only chromosome 13 displayed significantly increased number of genes with increased expression in Dukes D tumors compared to Dukes A–C tumors (*P* < 0.01).

### microRNA expression

#### Tumor tissue vs. normal colon tissue

There was no relationship between tumor stage and the number of differentially expressed microRNAs (Fig. 3c). Dukes A, B, C and D tumors showed 17%, 21%, 18% and 15% respectively of microRNAs with altered expression (FC > 0.5, FDR < 0.05) compared to normal colon tissue (Table 4). 173 microRNAs showed significantly altered expression in one or several combinations of Dukes stages and 55 microRNAs were altered in all Dukes groups located on chromosomes 1–9, 11, 13, 17–20 and 22. Six microRNAs showed significant changes in expression between Dukes A plus B vs. Dukes C plus D stages (Table 5).

### Combined statistical analyses of DNA and RNA alterations

#### Genome-wide interactions

Each Dukes tumor stage showed some genome wide statistical interactions between structural and transcriptional alterations (Fig. 3b), but only Dukes C and D tumors showed interactions accounting for DNA alterations that discriminated significantly between early (A plus B) and late (C plus D) tumors (Table 6). Altogether, 29% (6498/22094) of all genes had significant copy number changes or showed significantly altered expression in one or several combinations in Dukes A, B, C and D tumors. 1231 of these genes (19%, 1231/6498) showed chromosomal alterations in all four Dukes A–D stages and 406 genes (6%, 406/6498) showed combined interactions in the same direction (i.e. gain and upregulation or loss and downregulation).

### Chromosomal interactions

Chromosomes 4, 8, 13 and 20 displayed significant interactions between copy number changes and genes with significantly altered expression when isolated chromosomes were tested separately. The number of chromosomes with significant within-interactions increased with tumor progression according to Dukes stage; Dukes A showed one interaction and Dukes D 4 interactions.

23 microRNAs were located within altered DNA segments in Dukes A, B, C and D on chromosomes 1, 4, 7–9, 13, 17, 18 and 20 with 3, 3, 16 and 16 microRNAs altered in Dukes A, B, C and D respectively. One microRNA (microR-663 at 20p11.1) showed interactions with altered DNA sequences in all Dukes A–D tumor stages. All interacting microRNAs in Dukes A and B were present in Dukes C and D tumors, which imply that alterations in microRNA may be an early tumor phenomenon.

### Segmental interactions

The number of significant segmental interactions increased with tumor progression as illustrated for chromosome 8 (Fig. 2). Dukes A comprised 3 segments (66 Mb), Dukes B 3 (23 Mb), Dukes C 5 (358 Mb) and Dukes D 7 segments (244 Mb) with interactions between DNA and RNA. Three segments on chromosomes 8p and 18q showed interactions between DNA segments with loss and downregulation of expression. Eight regions at chromosome 7p/q, 8q, 13q and 20p/q showed interactions between DNA segments with copy number gain and upregulation.

#### Genes assumed important for carcinogenesis and tumor progression

Sixteen genes with significant mathematical interaction and upregulation were found in all Dukes tumors and were all located on chromosome 20. The DNA segment covered 40 Mb on chromosome 20p11.21– 20q13.33. These genes represented 0.2% of the total number of structurally altered genes on all chromosomes and may be relevant for the appearance of malignancy.

Genome wide DNA segment alterations with mathematical interaction to gene expression contained all together 41 genes with significantly altered expression in a manner that statistically discriminated between early (Dukes A plus B) versus late (Dukes C plus D) tumors (not shown); 28 of these genes were expressed in Dukes C plus D tumors and 17 in Dukes D tumors and may thus be relevant for tumor progression (Table 6). Ten of these genes (WDR67, RFXAP, RP11-50D16.3, CAB39L, THSD1, SPRY2, TGDS, CLDN10, SLC10A2, CD33L3) have been reported changed in tumor tissues, while only 2 (RP11-50D16.3, SLC10A2) have been reported to appear changed in colorectal cancer.

## Discussion

Technology progress in cancer research has been extraordinary with generation of enormous amounts of information particularly related to genomic and epigenetic alterations. Therefore, it appears more or less unlikely that it is possible to describe isolated and well defined causes behind appearance of malignant transformation or progression of cancer. It is easily recognized that combined alterations in gene structure, expression and processing of genetic information and epigenetic control of regulatory elements, may represent an infinite number of alterations in ranking critical events related to clinical outcome. Therefore, in the present study we used surrogate markers for outcome such as well established Dukes tumor stage classification of colon carcinoma in purposely a small group of individuals selected by chance as applied by others,^18^ since the relationship between Dukes stage and survival is well established worldwide. We combined DNA, RNA and microRNA arrays to identify tumor specific DNA copy number changes in relationship to early (Dukes A plus B) and late (Dukes C plus D) tumors. Tumor material and normal mucosa were all taken from the same individuals and genomic DNA and total RNA were processed from the same piece of tissue specimens. Statistical interaction analyses were based on DNA segments defined aberrant by DNA copy algorithm with subsequent determination of correlations to defined genes or transcripts with either significantly altered expression or content of tissue mRNA or microRNA. Pooled patient materials were intentionally used to stabilize for inter specimens variation, which enhances specificity but limits sensitivity in testing.

DNA sequence alterations in general and in early and late tumor stages agreed with our previous findings, where we used tiling BAC arrays to sub classify DNA sequence alterations in patients selected according to long and short term survival.^6^ Frequent early stage DNA changes included gains on chromosome 20 and parts of chromosomes 7p and 13q and loss in parts of chromosome 18q, while late tumor stages included gains of 7p, 7q, 8q, 13q and loss of 8p, 18p and 21q, suggesting great complexity within specific chromosomes as reported by others.^1^ Structural DNA and RNA alterations, interacting statistically significantly, increased from early to late tumor stages at both chromosomal, sub-chromosomal and gene levels. Also, interactions between DNA and microRNA increased significantly at gene levels in a similar way across Dukes A–D tumors. Chromosome 20 showed interaction between DNA and RNA in all Dukes A, B, C and D stages with MCR across all tumor stages. Thus, 40% of the aberrant bases in 3 out of 4 Dukes groups were located on chromosome 20, which makes it likely related to carcinogenesis and early invasiveness.^19^ DNA alterations on chromosome 20 have been reported by others indicating correlations between gains and transition from colon adenoma to carcinoma.^20^–^23^ Among altered genes on chromosome 20 in the present study were AURKA and CSE1L, which were also reported by others related to colorectal cancer.^23^,^24^ Thus, our results and conclusions agreed with findings reported by others based on genomic and transcriptomic information from different sources and patients,^18^ when our computations were performed on specific chromosomes. However, a different pattern appeared when early versus late tumor stages were used as covariates; then it appeared that chromosomes 13 and 18 were most important for transcriptional alterations due to changed DNA.

Copy number changes in DNA may reflect a natural adaptation of DNA to altered environmental conditions. This phenomenon may represent selections in development of life based on genetic recombination. Thus, cellular DNA that contains polymorphic regions may or may not represent future blue prints for improved functions. Based on this implication, it is not easy to judge what appearance of altered DNA sequences really imply in cells overriding contact inhibition and normal growth control including attenuated apoptosis. Such altered DNA structures may either represent appearing suitable adaptations to withstand hypoxia and other challenges; or it may only be a result of by chance events leading to further compromised cell function and growth control. A third explanation may be appearance of significant alterations without any impact at all on cell function; i.e. cells can continue to accumulate aberrant DNA as long as it does not compromise cell survival. However, DNA alterations important for carcinogenesis should be present in all subsequent tumor stages or tumor cell clones as long as the malignant cell remains. Late appearing DNA alterations may thus either imply changes determining tumor progression or simply that such changes are not destabilizing the genome too much. Therefore, a simplistic interpretation of our model approach was to discriminate and correlate early and late DNA copy number changes to statistically significant alterations in gene expression. This approach should exclude most structural DNA changes that are not translated into functional dynamics. Therefore, candidate DNA regions with interactions should contain a majority of copy number changes that could potentially influence on defined cellular functions by splicing and either increased or decreased translation. However, this simplistic approach would not identify DNA alterations that are related to as yet unconfirmed changes in gene expression. With this perspective it was also interesting to evaluate significant statistical interactions between microRNA and DNA copy number change, which may identify important interactions based on more recent dimensions of gene expression.

Genome-wide chromosomal copy number gain represented the only structural change that alone predicted progressive malignancy. Three genes showed inverse relationships between DNA structure and expression; i.e. gain and downregulation or loss and upregulation; a kind of combined alterations that make them less likely as functional adaptations. Also, we observed that altered expression in early stage tumors could disappear in later tumor stages, probably as a consequence of DNA loss. A majority of 28 genes with altered expression in Dukes C and D appeared to code for proteins in translation- and transcription control, cell transporting, membrane protein interactions and posttranslational modifications, although some genes had more or less unknown functions. Differentially expressed genes in Dukes A and B tumors did not correlate to confirmed aberrant DNA copy numbers (Table 6). A large proportion of genes with significantly altered expression and DNA interactions mapped to chromosome 13 (17/28), but 35% of these genes had unknown function. Our results indicated a clear-cut relationship between increasing number of combined genetic events (DNA and RNA or DNA and microRNA) and late Dukes stage, when we used a relative wide selection criteria for microRNA (FC < 0.5). However, as few as 6 microRNAs (including microR-602) were altered to discriminate between Dukes A and B versus Dukes C and D respectively. Only 4 microRNA genes were altered in Dukes A and B but not in C and D indicating few differences in microRNA between early and late tumor progression, although it has been reported that microR-602 and microR-373 may impact on systemic tumor spread. Accordingly, microR-373 was recently suggested a promoter of metastasis in breast cancer cells,^25^,^26^ now with similar indication in colon cancer. Upregulation of microR-21 was reported to correlate to poor outcome in colorectal cancer patients,^27^ but we did not find such implication in our present analysis accounting for tumor stage (Dukes A to D). Indeed, a lot of clinical and prognostic information appears to be confined to altered microRNAs in colon cancer,^28^ but such alterations seemed indirectly less related to DNA copy number changes since similar findings occurred in embryonic and transformed cells.^29^ Such observations agree with findings appearing in our present modeling. Only one of these six microRNAs (microR-486) was found to have a predicted target gene (CLDN10, Table 6, TargetScanHuman, the microR-Ontology Database), when search was performed among top 100 predicted target genes. Our observations on deregulated expressed microRNAs agreed to 70%–80% with selected sets of microRNAs from published reports.^30^–^33^

In conclusion, our present and previous observations indicate thousands of aberrant DNA copy numbers in genome wide analysis on colon cancer as expected.^6^,^7^,^34^ These numerous altered segments with potential importance for tumor progression were filtered by means of mathematical interaction analysis to a final group of 17 candidate genes (Dukes D) with hypothetical relevance for tumor progression. Our modeling supports that colon cancer progression is related to genomic instability accompanied by altered gene expression. However, new information is that carcinogenesis and early appearance of invasive tumor growth may rather be related to functional genomic alterations and less to DNA copy number changes. Our model may be a tool to accept or reject structural and functional genetic alterations in appearance and progression of colorectal cancer in small groups of patients.

## Figures and Tables

**Table 1.** Included patients operated on for primary colon carcinoma.

**Tumor stage**	**Gender**	**Age**	**Tumor localization**
**Dukes A**			
	M	82	Right colon
	M	75	Right colon
	M	75	Left colon
	F	66	Left colon
	F	75	Left colon
	F	81	Left colon
**Dukes B**			
	F	80	Right colon
	F	78	Left colon
	F	60	Left colon
	M	63	Left colon
	M	79	Left colon
	M	73	Left colon
**Dukes C**			
	M	76	Right colon
	F	86	Right colon
	F	55	Right colon
	F	72	Left colon
	M	73	Left colon
	M	62	Left colon
**Dukes D**			
	M	59	Right colon
	M	66	Right colon
	M	61	Left colon
	F	69	Left colon
	M	73	Left colon
	F	52	Left colon

All patients were consecutively included from a large cohort selected by chance over time.

**Table 2.** Copy number gain and loss in CGH analysis across Dukes A–D tumors compared to normal colon tissue from the same patients.

**Chromosome**	**Dukes tumor stage**								**No of bases**
	**A**	**B**	**C**	**D**	**A**	**B**	**C**	**D**	
	
	**Gain**				**Loss**				
1	0	0	0	195	0	0	37946	0	247179
2	0	0	2512	0	0	0	0	0	242690
3	0	0	0	0	0	0	1298	0	199288
4	0	0	0	0	0	0	182616	0	191121
5	0	0	0	0	0	1219	25902	0	180617
6	0	224	0	0	0	0	0	0	170734
7	56564	0	4919	77519	0	0	0	0	158781
8	678	0	104396	80270	0	0	0	31707	146251
9	0	0	0	2999	0	0	0	0	140129
10	0	0	0	0	0	0	824	0	135254
11	0	0	496	25	0	0	0	0	133951
12	0	0	0	0	0	0	0	0	132278
13	3440	8393	78167	95475	0	0	0	0	114077
14	0	0	0	0	0	0	0	3261	106330
15	0	0	0	83	0	0	0	1199	100169
16	0	0	5288	0	0	128	0	0	88652
17	0	0	0	0	0	0	975	21956	78623
18	0	0	0	0	0	58430	75913	75714	76083
19	0	0	0	0	0	0	0	0	63784
20	62345	39471	45970	42083	0	0	0	0	62364
21	0	0	0	0	0	0	21063	19817	46892
22	0	0	0	0	0	0	0	0	49525
TOT	123027	48088	241748	298649	0	59777	346537	153654	2864773

Gained or lost bases (kb) per chromosome among Dukes tumor stages were detected by DNA copy segment algorithm. Significant thresholds were specified by the 80th and 20th percentile respectively.

**Table 3.** Number of transcripts with significantly altered RNA expression in genome wide analyses of Dukes A–D tumors compared to normal colon tissue from the same patients.

**Chromosome**	**Dukes tumor stage**								**No of transcripts**
	**A**	**B**	**C**	**D**	**A**	**B**	**C**	**D**	
	
	**Upregulation**				**Downregulation**				
1	55	100	121	122	161	227	209	125	4046
2	64	101	117	94	119	148	115	73	2811
3	39	58	68	46	106	132	92	79	2293
4	44	65	71	53	83	114	86	73	1674
5	37	57	61	46	74	91	69	55	1978
6	54	81	78	58	61	85	114	50	2200
7	73	74	82	76	65	84	59	56	2090
8	44	69	83	57	45	74	38	42	1536
9	28	43	43	50	73	94	82	45	1624
10	32	48	42	57	62	95	66	51	1641
11	55	68	98	55	94	116	108	64	2178
12	49	70	77	54	84	91	90	65	2075
13	24	41	40	93	9	14	4	2	775
14	22	42	40	17	72	68	61	49	1301
15	32	39	47	32	67	77	66	55	1273
16	38	69	51	34	89	75	88	62	1706
17	41	65	57	33	91	107	114	92	2226
18	8	13	20	7	32	43	33	33	665
19	35	66	57	63	91	105	98	61	2338
20	58	118	73	58	34	42	32	28	1055
21	10	17	9	12	16	16	22	9	482
22	15	18	14	13	58	68	55	47	929
TOT	857	1322	1349	1130	1586	1966	1701	1216	38896

Transcription was considered significantly altered with log fold change >1 and adjusted *p*-value (FDR) <0.05 in total RNA from tumor tissue versus normal colon tissue.

**Table 4.** Number of micro RNAs in tumor tissue with significantly altered expression in genome wide analyses among Dukes A–D tumors compared to normal colon tissue from the same patients.

**Chromosome**	**Dukes tumor stage**								**No of probes**
	**A**	**B**	**C**	**D**	**A**	**B**	**C**	**D**	
	
	**Upregulation**				**Downregulation**				
1	11	18	16	6	17	19	29	17	96
2	0	1	0	0	8	7	6	7	38
3	3	6	0	2	6	5	8	5	58
4	5	4	2	5	5	5	3	4	51
5	4	3	2	0	9	12	8	8	44
6	0	0	0	0	6	6	5	5	31
7	16	17	15	11	2	2	6	2	64
8	0	0	0	0	5	10	5	2	38
9	4	6	9	7	10	6	3	8	43
10	–	2	1	–	–	0	0	–	38
11	4	2	4	2	8	9	4	8	37
12	1	2	4	–	0	0	2	–	45
13	16	17	17	17	0	0	2	0	32
14	8	16	8	1	5	1	4	11	148
15	1	4	4	8	0	4	0	0	34
16	0	4	0	0	2	6	4	2	22
17	3	4	5	3	10	16	6	8	69
18	0	0	0	0	3	3	3	3	8
19	3	0	7	3	8	9	4	6	170
20	3	3	1	3	0	0	0	0	23
21	2	3	2	0	3	3	0	5	12
22	3	3	2	3	0	0	0	0	24
TOT	87	115	99	71	107	123	102	101	1125

**Table 5.** microRNAs with significantly altered expression among early (Dukes A plus B) and late tumors (Dukes C plus D).

**Transcript**	**Regulation (A + B); (C + D)**		**Location**	**Found in**		
				**Solid tumors^a^**	**CRC**	**Hematological cancer**
miR-425–5p	↑	–	3p21.31	Yes	–	Yes
miR-625	↑	–	14q23.3	Yes		Yes
miR-144	↓	–	17q11.2	Yes		Yes
miR-486	↓	–	8p11.21	Yes^b^		–
miR-602	–	↑	9q34.3			
miR-373	–	↑	19q13.41	Yes	Yes^a^,^c^	

↑ Upregulation ↓Downregulation—Lack of significant change in expression between tumor and normal colon mucosa.

a miRÒ, The miR-Ontology Database.

b Mees, ST et al. Involvement of CD40 targeting miR-224 and miR-486 on the progression of pancreatic ductal adenocarcinomas. Ann Surg Oncol 16:2339-50, 2009.

c Monzo, M et al. Overlapping expression of microRNAs in human embryonic colon and colorectal cancer. *Cell Res*. 2008; 18:823–33.

**Table 6.** Transcripts (mRNA) with significantly altered expression located within DNA segments with significant copy number change in progressive colorectal tumors (Dukes C plus D versus Dukes A plus B).

**Gene**	**Location**	**Alterations in**			**References**		**Systemic name mRNA (NM_)**
		**DNA**	**RNA**	**Protein function**	**E**	**F**	
**Dukes A**							
**Dukes A–C**							
**Dukes C–D**							
STX1 A	7q11.23	↑	↑	Transport			004603.2
CLDN4	7q11.23	↑	↓	Membrane, Development	(35, 36)	(35)	001305.3
CLDN3	7q11.23	↑	↓	Membrane, Development	(35, 36)	(35)	001306.3
RPL30	8q22.2	↑	↑	Ribosome	(37)	(37)	000989.2
PABPC1	8q22.3	↑	↑	Translation initiation	(38, 39)	–	002568.3
TATDN1	8q24.13	↑	↑	Hepatocarcinoma	(40)	–	032026.2
EXOSC8	13q13.3	↑	↑	RNA processing	–	–	181503.2
C13orf7 (RNF219)	13q31.1	↑	↑	Unknown function	–	–	024546.3
RANBP5 (IP05)	13q32.2	↑	↑	Transport	–	–	002271.4
TPP2	13q33.1	↑	↑	Proteolys	–	–	003291.2
SLC14A1–002	18q12.3	↑	↑	Transport	–	–	001128588.1
**Dukes D**							
WDR67	8q24.13	↑	↑	Unknown function	(41)	–	001145088.1
RFXAP	13q13.3	↑	↑	Transcription factor	(42, 43)	–	000538.3
ALG5	13q13.3	↑	↑	Glycosylation	–	–	001142364.1
RP11-50D16.3 (NHLRC3)	13q13.3	↑	↑	Unknown function	(44)	(45)	001012754.2
KIAA1704	13q14.12	↑	↑	Unknown function	–	–	018559.2
CAB39 L	13q14.2	↑	↑	Calcium binding protein	(46)	–	030925.2
THSD1	13q14.3	↑	↑	Extracellular matrix	(47)	–	018676.3
AL831999	13q14.3	↑	↑	Unknown function	–	–	
SPRY2	13q31.1	↑	↑	Signaling	(48–50)	–	005842.2
TGDS	13q32.1	↑	↑	Glucose dehydratase	(51)	–	014305.2
CLDN10	13q32.1	↑	↑	Membrane-tight junction	(36, 52)	–	182848.2
SLC10A2	13q33.1	↑	↓	Transport-sodium/bile acid	(53, 54)	(53, 54)	000452.2
ANKRD10	13q34	↑	↑	Unknown function	–	–	017664.2
PCID2	13q34	↑	↑	Unknown function	–	–	018386
AF263545	18q12.3	↓	↑	Unknown function	–	–	(AF263545)
CD33 L3 (SIGLEC15)	18q12.3	↓	↑	Membrane	(55)	–	213602.1
ATP5A1	18q21.1	↓	↓	Transport	–	–	001001937.1

Genes with unknown function have been reported.^56^

**Abbreviations:** E, Earlier reported in human malignant disease; F, Earlier reported in human colon cancer; ↑Copy number gain (DNA); increased expression (RNA); ↓Copy number loss (DNA); decreased expression (RNA).

## References

[1] Frohling S Dohner H Chromosomal abnormalities in cancer N Engl J Med 2008 359 7 722 34 18703475 10.1056/NEJMra0803109

[2] Timp W Levchenko A Feinberg AP A new link between epigenetic progenitor lesions in cancer and the dynamics of signal transduction Cell Cycle 2009 8 3 383 90 19177016 10.4161/cc.8.3.7542 PMC6275123

[3] Irizarry RA Ladd-Acosta C Wen B The human colon cancer methylome shows similar hypo- and hypermethylation at conserved tissuespecific CpG island shores Nat Genet 2009 41 2 178 86 19151715 10.1038/ng.298 PMC2729128

[4] Nephew KP Huang TH Epigenetic gene silencing in cancer initiation and progression Cancer Lett 2003 190 2 125 33 12565166 10.1016/s0304-3835(02)00511-6

[5] Yang L Belaguli N Berger DH MicroRNA and Colorectal Cancer World J Surg 2009 10.1007/s00268-008-9865-5 19123024

[6] Lagerstedt K Staaf J Jönsson G Tumor genome wide DNA alterations assessed by array CGH in patients with poor and excellent survival following operation for colorectal cancer Cancer Informatics 2007 3 1 15 19455253 PMC2675850

[7] Markowitz SD Bertagnolli MM Molecular origins of cancer: Molecular basis of colorectal cancer N Engl J Med 2009 361 25 2449 60 20018966 10.1056/NEJMra0804588 PMC2843693

[8] Ley TJ Mardis ER Ding L DNA sequencing of a cytogenetically normal acute myeloid leukaemia genome Nature 2008 456 7218 66 72 18987736 10.1038/nature07485 PMC2603574

[9] Stratton MR Campbell PJ Futreal PA The cancer genome Nature 2009 458 7239 719 24 19360079 10.1038/nature07943 PMC2821689

[10] R Development Core Team, R Language and Environment for Statistical Computing 2009

[11] Gentleman RC Carey VJ Bates DM Bioconductor: open software development for computational biology and bioinformatics Genome Biol 2004 5 10 R80 15461798 10.1186/gb-2004-5-10-r80 PMC545600

[12] Olshen AB Venkatraman ES Lucito R Wigler M Circular binary segmentation for the analysis of array-based DNA copy number data Biostatistics 2004 5 4 557 72 15475419 10.1093/biostatistics/kxh008

[13] Aguirre AJ Brennan C Bailey G High-resolution characterization of the pancreatic adenocarcinoma genome Proc Natl Acad Sci U S A 2004 101 24 9067 72 15199222 10.1073/pnas.0402932101 PMC428474

[14] Yang YH Dudoit S Luu P Normalization for cDNA microarray data: a robust composite method addressing single and multiple slide systematic variation Nucleic Acids Res 2002 30 4 e15 11842121 10.1093/nar/30.4.e15 PMC100354

[15] Smyth GK Linear models and empirical bayes methods for assessing differential expression in microarray experiments Stat Appl Genet Mol Biol 2004 3 Article3. 10.2202/1544-6115.1027 16646809

[16] Benjamini Y Hochberg Y Controlling the false discovery rate: A practical and powerful approach to multiple testing J Royal Statistical Soc 1995 57 1 289 300

[17] Bolstad BM Irizarry RA Astrand M Speed TP A comparison of normalization methods for high density oligonucleotide array data based on variance and bias Bioinformatics 2003 19 2 185 93 12538238 10.1093/bioinformatics/19.2.185

[18] Tsafrir D Bacolod M Selvanayagam Z Relationship of gene expression and chromosomal abnormalities in colorectal cancer Cancer Res 2006 66 4 2129 37 16489013 10.1158/0008-5472.CAN-05-2569

[19] Habermann JK Paulsen U Roblick UJ Stage-specific alterations of the genome, transcriptome, and proteome during colorectal carcinogenesis Genes Chromosomes Cancer 2007 46 1 10 26 17044061 10.1002/gcc.20382

[20] Hermsen M Postma C Baak J Colorectal adenoma to carcinoma progression follows multiple pathways of chromosomal instability Gastroenterology 2002 123 4 1109 19 12360473 10.1053/gast.2002.36051

[21] Carvalho B Postma C Mongera S Multiple putative oncogenes at the chromosome 20q amplicon contribute to colorectal adenoma to carcinoma progression Gut 2009 58 1 79 89 18829976 10.1136/gut.2007.143065

[22] Bertucci F Salas S Eysteries S Gene expression profiling of colon cancer by DNA microarrays and correlation with histoclinical parameters Oncogene 2004 23 7 1377 91 14973550 10.1038/sj.onc.1207262

[23] Camps J Grade M Nguyen QT Hormann P Becker S Hummon AB Chromosomal breakpoints in primary colon cancer cluster at sites of structural variants in the genome Cancer Res 2008 68 5 1284 95 18316590 10.1158/0008-5472.CAN-07-2864 PMC4729303

[24] Bertucci F Finetti P Rougemont J Charafe-Jauffret E Nasser V Loriod B Gene expression profiling for molecular characterization of inflammatory breast cancer and prediction of response to chemotherapy Cancer Res 2004 64 23 8558 65 15574762 10.1158/0008-5472.CAN-04-2696

[25] Huang Q Gumireddy K Schrier M le Sage C Nagel R Nair S The microRNAs miR-373 and miR-520c promote tumour invasion and metastasis Nat Cell Biol 2008 10 2 202 10 18193036 10.1038/ncb1681

[26] Negrini M Calin GA Breast cancer metastasis: a microRNA story Breast Cancer Res 2008 10 2 203 18373886 10.1186/bcr1867 PMC2397516

[27] Schetter AJ Leung SY Sohn JJ Zanetti KA Bowman ED Yanaihara N MicroRNA expression profiles associated with prognosis and therapeutic outcome in colon adenocarcinoma JAMA 2008 299 4 425 36 18230780 10.1001/jama.299.4.425 PMC2614237

[28] Schepeler T Reinert JT Ostenfeld MS Christensen LL Silahtaroglu AN Dyrskjot L Diagnostic and prognostic microRNAs in stage II colon cancer Cancer Res 2008 68 15 6416 24 18676867 10.1158/0008-5472.CAN-07-6110

[29] Monzo M Navarro A Bandres E Artells R Moreno I Gel B Overlapping expression of microRNAs in human embryonic colon and colorectal cancer Cell Res 2008 18 8 823 33 18607389 10.1038/cr.2008.81

[30] Kim S Choi M Cho KH Identifying the target mRNAs of microRNAs in colorectal cancer Comput Biol Chem 2009 33 1 94 9 18723399 10.1016/j.compbiolchem.2008.07.016

[31] Bandres E Cubedo E Agirre X Malumbres R Zarate R Ramirez N Identification by Real-time PCR of 13 mature microRNAs differentially expressed in colorectal cancer and non-tumoral tissues Mol Cancer 2006 5 29 16854228 10.1186/1476-4598-5-29 PMC1550420

[32] Motoyama K Inoue H Takatsuno Y Tanaka F Mimori K Uetake H Over- and under-expressed microRNAs in human colorectal cancer Int J Oncol 2009 34 4 1069 75 19287964 10.3892/ijo_00000233

[33] Arndt GM Dossey L Cullen LM Lai A Druker R Eisbacher M Characterization of global microRNA expression reveals oncogenic potential of miR-145 in metastatic colorectal cancer BMC Cancer 2009 9 374 19843336 10.1186/1471-2407-9-374 PMC2770572

[34] Hoeijmakers JH DNA damage, aging, and cancer N Engl J Med 2009 361 15 1475 85 19812404 10.1056/NEJMra0804615

[35] Hewitt KJ Agarwal R Morin PJ The claudin gene family: expression in normal and neoplastic tissues BMC Cancer 2006 6 186 16836752 10.1186/1471-2407-6-186 PMC1538620

[36] Morin PJ Claudin proteins in human cancer: promising new targets for diagnosis and therapy Cancer Res 2005 65 21 9603 6 16266975 10.1158/0008-5472.CAN-05-2782

[37] Jiang W Li X Rao S Wang L Du L Li C Constructing disease-specific gene networks using pair-wise relevance metric: application to colon cancer identifies interleukin 8, desmin and enolase 1 as the central elements BMC Syst Biol 2008 2 72 18691435 10.1186/1752-0509-2-72 PMC2535780

[38] Liu Y Zhu X Zhu J Liao S Tang Q Liu K Identification of differential expression of genes in hepatocellular carcinoma by suppression subtractive hybridization combined cDNA microarray Oncol Rep 2007 18 4 943 51 17786358

[39] Takashima N Ishiguro H Kuwabara Y Kimura M Haruki N Ando T Expression and prognostic roles of PABPC1 in esophageal cancer: correlation with tumor progression and postoperative survival Oncol Rep 2006 15 3 667 71 16465428

[40] Carrasco DR Tonon G Huang Y Zhang Y Sinha R Feng B Highresolution genomic profiles define distinct clinico-pathogenetic subgroups of multiple myeloma patients Cancer Cell 2006 9 4 313 25 16616336 10.1016/j.ccr.2006.03.019

[41] Savola S Klami A Tripathi A Niini T Serra M Picci P Combined use of expression and CGH arrays pinpoints novel candidate genes in Ewing sarcoma family of tumors BMC Cancer 2009 9 17 19144156 10.1186/1471-2407-9-17 PMC2633345

[42] Cai B Hogg D Lu G Liu L Xi X Xu W Studies of differential-expressed genes in human endometrial cancer of various differentiated grades Chinese J Clin Oncol 2007 4 2 77 82

[43] Lancaster JM Dressman HK Pittman J Gray J Sayer R West M Identification of genes assosiated with ovarian cancer metastasis using microarray expression analysis Int J Gyn Cancer 2008 14 Suppl 1 24 10.1111/j.1525-1438.2006.00660.x 17009964

[44] Finch A Metcalfe K Lui J Springate C Demsky R Armel S Breast and ovarian cancer risk perception after prophylactic salpingo-oophorectomy due to an inherited mutation in the BRCA1 or BRCA2 gene Clin Genet 2009 75 3 220 4 19263514 10.1111/j.1399-0004.2008.01117.x

[45] Daley D Lewis S Platzer P MacMillen M Willis J Elston RC Identification of susceptibility genes for cancer in a genome-wide scan: results from the colon neoplasia sibling study Am J Hum Genet 2008 82 3 723 36 18313025 10.1016/j.ajhg.2008.01.007 PMC2427227

[46] Zhang PY Zhang WG He AL Wang JL Li WB Identification and functional characterization of the novel acute monocytic leukemia associated antigen MLAA-34 Cancer Immunol Immunother 2009 58 2 281 90 18592235 10.1007/s00262-008-0552-z PMC11030758

[47] Ko JM Chan PL Yau WL Chan HK Chan KC Yu ZY Monochromosome transfer and microarray analysis identify a critical tumor-suppressive region mapping to chromosome 13q14 and THSD1 in esophageal carcinoma Mol Cancer Res 2008 6 4 592 603 18403638 10.1158/1541-7786.MCR-07-0154

[48] Frank MJ Dawson DW Bensinger SJ Hong JS Knosp WM Xu L Expression of sprouty2 inhibits B-cell proliferation and is epigenetically silenced in mouse and human B-cell lymphomas Blood 2009 10.1182/blood-2008-05-156943 PMC2656273 19147787

[49] Fritzsche S Kenzelmann M Hoffmann MJ Muller M Engers R Grone HJ Concomitant down-regulation of SPRY1 and SPRY2 in prostate carcinoma Endocr Relat Cancer 2006 13 3 839 49 16954433 10.1677/erc.1.01190

[50] Tsavachidou D Coleman ML Athanasiadis G Li S Licht JD Olson MF SPRY2 is an inhibitor of the ras/extracellular signal-regulated kinase pathway in melanocytes and melanoma cells with wild-type BRAF but not with the V599E mutant Cancer Res 2004 64 16 5556 9 15313890 10.1158/0008-5472.CAN-04-1669

[51] Du JW Gu JY Liu J Cen XN Zhang Y Ou Y TCR spectratyping revealed T lymphocytes associated with graft-versus-host disease after allogeneic hematopoietic stem cell transplantation Leuk Lymphoma 2007 48 8 1618 27 17701594 10.1080/10428190701474357

[52] Sun B Wu J Zhang T Wang C High-resolution analysis of genomic profiles of hepatocellular carcinoma cells with differential osteopontin expression Cancer Biol Ther 2008 7 3 387 91 18717013 10.4161/cbt.7.3.5365

[53] Grunhage F Jungck M Lamberti C Keppeler H Becker U Schulte-Witte H Effects of common haplotypes of the ileal sodium dependent bile acid transporter gene on the development of sporadic and familial colorectal cancer: a case control study BMC Med Genet 2008 9 70 18644122 10.1186/1471-2350-9-70 PMC2492852

[54] Anderle P Sengstag T Mutch DM Rumbo M Praz V Mansourian R Changes in the transcriptional profile of transporters in the intestine along the anterior-posterior and crypt-villus axes BMC Genomics 2005 6 1 69 15882471 10.1186/1471-2164-6-69 PMC1145182

[55] Smith SC Nicholson B Nitz M Frierson HF Jr Smolkin M Profiling bladder cancer organ site-specific metastasis identifies LAMC2 as a novel biomarker of hematogenous dissemination Am J Pathol 2009 174 2 371 9 19147813 10.2353/ajpath.2009.080538 PMC2630547

[56] Strausberg RL Feingold EA Grouse LH Generation and initial analysis of more than 15,000 full-length human and mouse cDNA sequences Proc Natl Acad Sci U S A 2002 99 26 16899 903 12477932 10.1073/pnas.242603899 PMC139241

